# "You don’t get side effects from social prescribing”—A qualitative study exploring community pharmacists’ attitudes to social prescribing

**DOI:** 10.1371/journal.pone.0301076

**Published:** 2024-05-16

**Authors:** Adam Pattison Rathbone, Harry Pearson, Oluwafunmi Akinyemi, Nia Cartwright, Stephanie Tierney, Gill Rowlands, Laura Lindsey

**Affiliations:** 1 Faculty of Medical Sciences, Newcastle University, Newcastle upon Tyne, United Kingdom; 2 Nuffield Department of Primary Care Science, University of Oxford, Oxford, United Kingdom; 3 Independent Research Pharmacist, United Kingdom; Keele University, UNITED KINGDOM

## Abstract

**Objectives:**

Social prescribing is an approach that enables the referral of patients to non-clinical support and places a focus on holistic care. This study explored views of community pharmacists regarding social prescribing in pharmacies.

**Study design:**

A qualitative phenomenological approach was used.

**Methods:**

A convenience sample of eleven community pharmacists from Northern England were recruited via social media (Twitter, Facebook) and took part in a semi-structured, one-to-one qualitative interviews that asked about their knowledge of social prescribing, the advantages of community pharmacist involvement and any barriers they predicted to its implementation. Interviews were transcribed verbatim and thematically analysed.

**Results:**

The sample included largely male pharmacists (63.3%) with less than five years’ experience (45.5%) and included pharmacists working as employees (63.6%), locums (27.3%) and owners (9%) in both chain (36%) and independent stores (54.5%). The main findings indicate an enthusiasm for but limited understanding of social prescribing. Factors which appeared to influence involvement were training requirements and time available to complete an additional service in busy pharmacies. Opportunities centred on the broader pharmacy team’s role to optimise health outcomes.

**Conclusions:**

The findings indicate pharmacists may be an underused resource due to a poor understanding of the full scale and scope of social prescribing beyond health promotion, lifestyle interventions. Further work is needed to explore the transferability of the findings to the broader pharmacy workforce to understand how social prescribing can be positioned within pharmacy practice.

## Introduction

Social prescribing has been described as: “*a means for health-care workers to connect patients to a range of non-clinical services in the community to improve health and well-being*. *Social prescribing can help to address the underlying causes of patients’ health and well-being issues*, *as opposed to simply treating symptoms*.” [1]. It has become a cornerstone of healthcare policy in the UK [2] and overseas [3]. Patients are often referred for social prescribing by a General Practitioner (GP), who contacts a link worker to meet with the patient to identify appropriate support for their non-medical needs [3]. Exercise classes, arts and crafts, volunteering, gardening, and cookery classes, as well as accommodation and debt management services, are among the types of support patients might be connected to by a link worker [4–6].

It is argued that social prescribing can be useful for people living with long-term health conditions, mental health problems, socioeconomic struggles and social isolation–the latter of which has become more prevalent following the COVID-19 pandemic [7]. There is some evidence suggesting engagement in social prescribing may reduce demand for non-elective healthcare [8] and GP attendances [9]. Hence, it may be a means of addressing demand on overstretched healthcare services, supporting the broader well-being of vulnerable, socioeconomically disadvantaged communities. However, it should be noted, claims social prescribing reduces health inequalities for socioeconomically deprived communities are still considered contentious and their impact on reducing demand of some healthcare services, such as pharmacies, is not known [10–13].

Approaches to expanding access to social prescribing are being explored in the UK through ‘proactive social prescribing’ [14], where patient populations are screened by primary care professionals to identify target groups with unmet needs. Other examples of improving access to social prescribing are schemes such as digital self-referrals, where an app matches patients with appropriate support in the community [15]. In addition, an appeal for further healthcare professionals, including pharmacists, to take a role in social prescribing has been made [16].

Pharmacies offer open access to healthcare for a wide range of people, in both rural and urban settings. Pharmacies are known to be more accessible than GPs in areas of high socioeconomic deprivation [17]. Evidence suggests during the pandemic in the UK in 2020, over a third of patients visited their community pharmacy instead of their GP practice [18] although it was unclear if this prevented follow-up visits to GP practices. Although pharmacies are reporting high workloads [19–21], their accessibility makes them suitable for healthcare initiatives to reduce demand on existing health services [15, 17]. Despite this, funding for pharmacies in the UK is focused primarily on dispensing medications rather than patient-facing services [22]. Recent changes to policy, such as the NHS Long Term Plan, Healthy Living Pharmacy Framework, Pharmacy First and the Community Pharmacy Consultation Scheme, indicate pharmacies will be an increasingly important place for the delivery of healthcare services, both urgent and preventative, in the future [2, 23–26].

The evidence evaluating social prescribing interventions in pharmacy is limited. A systematic review in 2017 found little evidence of the efficacy of social prescribing in community settings, due to the short-term nature of the evaluations [27]. Other work focusing specifically on pharmacies found similarly limited literature [28]. To improve the existing evidence for social prescribing in pharmacy, evaluations must start from the foundations and work up; identifying capabilities, opportunities and motivations of pharmacy teams as well as the impact on patients, community groups and other health and social care professionals in the system. Little is known about pharmacists’ awareness and understanding of social prescribing and what factors influence their involvement in this non-clinical activity [28, 29]. What evidence does exist suggests workload and funding may limit the involvement of pharmacists, and that these professionals may have limited awareness of what social prescribing is [29]. This existing evidence is based on quantitative methods and, thus, did not provide a detailed description of pharmacists’ experiences of social prescribing in practice. Hence, the aim of our study was to explore community pharmacists’ experiences, perspectives and attitudes to social prescribing in practice.

## Methods

A qualitative phenomenological approach was adopted, which drew on the Capability Opportunity Motivation–Behaviour (COM-B) model [30]. A phenomenological approach allowed the study to focus on community pharmacists’ experiences of what happens in practice and how it happens [31]. Specifically, the COM-B model was used to create a topic guide to use during interviews and added structure to the findings following the identification of themes. As evidence in relation to pharmacists’ roles in social prescribing is limited, an exploratory design was appropriate [30].

### Participants and recruitment

A convenience sampling method was adopted. A form was posted on social media (Twitter and Facebook) to allow participants to self-screen against inclusion criteria. The criteria included having experience working as a community pharmacist in Northern England, conversant in English, and capacity to consent to research. Snowball sampling was also used to recruit participants to the study.

The form was created and posted on social media via the research team (APR, HP, LL) outlining the study. Users completed screening questions for eligibility and were prompted to give an email address and telephone number to be contacted. The decision to limit to Northern England was pragmatic as researchers were based there. It also allowed the study to recruit pharmacists practicing in areas of high deprivation, where pharmacists are known to be more readily accessed by patients than in areas of low deprivation [17]. A participant information sheet was provided to interviewees in advance, and verbal consent was taken prior to participation, which was recorded by the interviewer (HP) and witnessed by the supervisor (APR).

### Methods of data collection

Semi-structured interviews were conducted via the online platforms Zoom and Microsoft Teams, and over the telephone between Monday 5th October 2020 and Friday 29^th^ January 2021. The semi-structured nature of the interviews allowed for an in-depth exploration of pharmacists’ views, which would be unobtainable via a survey [32]. Interviews were conducted at a time convenient to the participant. Interviews lasted between 30 and 45 minutes (average = 39 minutes). A topic guide was used (see Tables 1 and 2) that included questions such as: i) What are your experiences of social prescribing? ii) What do you understand as the advantages of social prescribing in community pharmacy? iii) What do you think are the barriers to implementing social prescribing in community pharmacy?

**Table 1:** Summary of participant demographics.

Description	N	%
**Total**	**11**	**100**
**Sex**		
Male	3	63.6
Female	8	27.3
Other	0	0
**Level of experience**		
Pharmacists with less experience (Post-registration Foundation Training)	5	45.5
Pharmacists with more experience (Advanced Pharmacist)	6	54.5
**Status**		
Owner	1	9.1
Employee	7	63.6
Locum	3	27.3
**Primarily experience of**		
Working in Independent stores	6	54.5
Working in Multiple Chains	4	36.4
Other*	1	9.1

* this included a pharmacist who worked in a pharmacy attached to a GP surgery

**Table 2:** Themes, clusters and coding structure.

Theme	Clusters	Codes
Varied knowledge and understanding of social prescribing	Knowledge and awareness Understanding of social prescribing activities	No awareness No knowledge Heard of it Healthy lifestyle consultations Social history taking Group activities Exercise classes Link to physical health and well-being Disease specific support
Factors influencing involvement in social prescribing	Workload Expectations Access to resources	Dispensing items Consultations Services Vaccinations Staff shortages High workload Increased patient facing roles Increased services COVID19 pandemic Timing Role of the pharmacist Patient fear Stigma Financial pressure Resources Corporate targets—money Customer loyalty–community Community versus commerce
Opportunities for social prescribing in community pharmacy	Pharmacy team An opportunity to improve patient care	Technicians Dispensers Delivery drivers GPs Pharmacists Training Patient outcomes Patient benefits

One-to-one interviews were conducted by a final year pharmacy student (HP). He was trained by experienced qualitative health researchers (LL, GR, APR). Interviews were audio-recorded and transcribed manually by one author (HP) with a 10% sample quality checked by listening back to the audio and reading the transcript (APR) [33, 34]. Transcripts were anonymised by removing the names of participants, people, and places [33, 34]. Data collection ceased at the point of theoretical data sufficiency [35], which occurred after ten interviews; one additional interview was conducted to confirm this was the case. Theoretical data sufficiency relates to the point in the study at which no new findings are being identified in the data. This was operationalised in the study through regular weekly supervision meetings during data collection and analysis to interrogate, explore and identify when no new findings were being found. This point indicates the research team had access to sufficient data to draw conclusions, though due to the nature of qualitative inquiry, further findings may be found by new researchers looking at the same data.

### Data processing and analysis

Transcripts were imported into NVivo and inductive thematic analysis was completed by three researchers (HP, APR, LL) using the method outlined by Braun and Clarke (33). The first step of this analysis began with familiarisation with the data, next there was generation of initial codes, then clusters were created, and finally themes. A constant comparative approach was adopted, which meant codes, clusters and themes were compared with one another and findings interrogated in data presentation meetings involving the authors. This process was underpinned by a phenomenological understanding of experiences, which focuses on what the essence of an experience is and how this happens–i.e., what was happening and how was it happening. The COM-B Model was then used to contextualise the themes to link what happened to behavioural theory.

As part of the analysis, participants were categorised based on their role within community pharmacy and the length of time since registration. Participants with experience of less than five years were classified as ‘pharmacists with less experience’. This classification is in line with The Royal Pharmaceutical Society (RPS) Foundation Pharmacist Framework [36]. Participants with experience greater than this were categorised as ‘pharmacists with more experience’. This follows the RPS Advanced Pharmacy Framework [37]. Credibility was defined as the ability of the findings of the study to be reasonably believed and dependability was defined as the ability to trust the research process was carried out accurately. Credibility and dependability were established by involving more than one person in the analysis (sometimes referred to as analytical triangulation), through presentation and discussion of data at regular coding meetings with the research team. Weekly meetings were also used to ensure senior authors (LL, GR, APR) provided suitable training, support, supervision and accountability to the team (HP, NC). The study processes and findings were also reviewed by external collaborators (OA, ST) which further enhanced the credibility and dependability of the findings.

### Reflexivity

Reflexivity allows research authors to become aware of, respond to and acknowledge how their own personal characteristics, identity and perspectives influences research [38]. In this study, the authors came from working class, middle class and upper middle-class backgrounds, where mostly White and came from Britain. Two authors were not from Britain and two authors were not White. Three authors were pharmacists, one was a general practitioner and one a psychologist. Four authors had completed, and one author was completing, a PhD. The research was led by a team based in a School of Pharmacy and this meant members of the research team may have been well known to participants as former educators (LL, APR) or colleagues (HP, OA). This meant there was a shared understanding of language and terminology between participants and researchers which enriched the subjectivity of the study during data collection. However, other members of the research team (NC, ST, GR) were less professionally connected to the pharmacy sector and so provided an objective perspective during data analysis and interpretation.

### Research ethics

Ethical approval for this study was obtained through Newcastle University (reference number 6162/2020).

## Results

### Participant demographics

Eleven participants were recruited and demographics are summarised in Table 1.

### Thematic findings

Findings are described below, with codes, clusters and themes shown in Table 2. Data extracts describe findings in participants’ own words. Quotes denote if participants were employee, locum or owner pharmacists and which ‘type’ of pharmacy they worked in–either an ‘independent’ pharmacy which refers to a small chain, local, or single pharmacy business or a ‘multiple’ pharmacy chain which refers to a large, multi-national pharmacy corporation with many pharmacy businesses operating under a single banner.

#### Theme 1) Varied knowledge and understanding of social prescribing

Most participants seemed to have an awareness of and enthusiasm for social prescribing, although they reported little knowledge of it. The setting where participants worked, their status or level of experience did not appear to influence knowledge or reported beliefs about social prescribing. Participants who were aware of social prescribing appeared to know about it either from involvement in a social prescribing event, through prescribing community-based, non-clinical support themselves, or having heard about the process in previous employment.

“*I was running group clinics…where we don’t just talk about their medicines, we talk about interventions like exercise, or lifestyle advice or diet. It was much more informal, but we would make recommendations to patients like a Tai Chi class for example, that you would benefit from, or you might be better off doing some core strengthening exercises given your type of arthritis.” Participant 6 (Pharmacist, Independent)*

Despite a limited understanding, pharmacists appeared to believe they had capability to support social prescribing. However, they appeared to view it as a clinician-led approach, focusing on the physical symptoms of disease, rather than a person-centred approach to address socioeconomic factors of health and well-being, directed by the patient to address their specific needs. This indicated social prescribing was being conflated with public health promotion, lifestyle campaigns.

“*I can really see where [social prescribing] would fit in that remit, so the kind of physical and the recommendation for physical activity, how it can help with a number of different medical conditions… we’ve got a really good knowledge base of different health conditions and generally kind of how the body works. So why not use that and I don’t think we’re using it a lot at the minute.” Participant 1 (Locum, Independent)*

These findings demonstrate the nuance of pharmacists’ approach to social prescribing, in that, enthusiasm toward social prescribing was reported, but that this appeared to be based on an understanding of social prescribing as an aspect of health promotion and lifestyle interventions based on physical disease states, rather than socio-economic circumstances of the patient.

#### Theme 2) Factors influencing involvement in social prescribing

Concern about the economics of a pharmacy businesses, the balance of workload and funding, was a recurring factor which appeared to shape participants’ thoughts about involvement in social prescribing. Participants reported the busy nature of community pharmacy and highlighted how much additional time would be needed to engage with social prescribing.

“*Well with the increase of both dispensing items, the more and more consultations that we are having to do, as well as the fact some stores due to cuts [to funding] have had to get rid of managers, that then falls on the pharmacist’s desk, there’s a lot less time for patient-pharmacist discussions. So timing is going to be a major issue I think.” Participant 11 (Employee, Multiple)*

Additional demands and additional time pressure, following on from the relentless experience encountered during the pandemic for many pharmacists through involvement in vaccination and increased workload, were concerns participants shared.

“*I certainly have worked over the COVID-19 pandemic in community myself, I know how ridiculously busy we’ve been. You know to try and fit in another additional service on top of all of the ones that are already being offered… but I think currently I’ve never known pharmacy this busy in my entire career…” Participant 6 (Employee, Independent)*

Hesitancy also related to patients’ responses to being offered social prescribing in a pharmacy setting.

“*They might feel embarrassed to accept that help. And they might find it quite intrusive, they might not expect a pharmacist to be involved and…nobody wants to be categorised as a vulnerable or isolated patient particularly.” Participant 4 (Employee, Multiple)*

Participants reported feeling that larger ‘multiples’ companies had greater resources and financial capital and would therefore find it easier to implement social prescribing services than independent organisations.

“*And the small pharmacies I worry that because they’re so, their [funding is] so tight they are trying to make the best they can being an independent that they won’t sort of have the capacity necessary to widen to some of these sorts of wider societal things that they can have input in.” Participant 5 (Employee, Multiple)*

Conversely, others suggested larger organisations with more capital may focus on profits rather than supporting patient communities, unlike independent organisations.

“*I’ve worked for [supermarket pharmacy 1] and [supermarket pharmacy 2] before which are bigger companies, and I know they’re much more focused around [funding]…rather than the kind of community support and health advice [in social prescribing]. I think an independent might do it because of the benefit to the community and to be seen to be giving extra services which might attract and keep their customer base.” Participant 6 (Employee, Independent)*

The only participant who was a pharmacy owner (and therefore responsible for organisational structure and financial targets of a community pharmacy business) reported social prescribing was an individual, professional decision of the pharmacist in charge, rather than the priorities of the business or owner. This appeared to diminish the role of organizational policy, working conditions (such as opening times and staffing levels), and the funding landscape, suggesting engagement with social prescribing will come down to personal preference of the individual pharmacist.

“*There’s no [funding] difference between the individual pharmacists, whoever they work for. So, under those circumstances it doesn’t matter if it’s an independent or a multiple pharmacy, they will organise themselves differently, but it’ll come down to the individuals, not the policy of the owner.” Participant 10 (Owner, Independent)*

Collectively, these findings indicate pharmacists’ motivations to deliver social prescribing services are influenced by access to appropriate levels of economic capital and resources to manage workload and patient expectations.

#### Theme 3) Outcomes of social prescribing in community pharmacy

This theme describes social prescribing as an opportunity for pharmacies to improve patient outcomes by involving all members of the pharmacy team, not just pharmacists. The inclusion of all staff into social prescribing was raised by participants. The knowledge and trust shared with patients was considered to make them a good resource to facilitate social prescribing. Participants felt dispensing staff, delivery drivers, and pharmacy technicians, as well as pharmacists, represented valuable assets to facilitate social prescribing, if given appropriate training and links to social prescribing networks.

Participants appeared to clearly understand the accessibility of pharmacy and highlighted the patient-centeredness of pharmacies, in comparison to other healthcare settings for patients, was aligned to social prescribing principles.

“*We are the most accessible healthcare professional in every community, and patients know they can just pop in for that source of advice. We have a lot more time [than other healthcare professionals] to tailor to individual patients” Participant 11 (Employee, Multiple)*

Participants reported valuing the role that social prescribing could play in improving health outcomes for patients, lessening the need for medication and expensive treatments.

“*…you’ve got the obvious benefits to the patients around outcomes…it might be that they are prescribed metformin for type 2 diabetes, which alongside social prescribing around diet and exercise…as a result of the diet and exercise intervention that the whole…type 2 diabetes will be better off.” Participant 5 (Employee, Multiple)*

Additionally, participants appeared to recognise opportunities to improve patient care by providing an alternative to medications.

“*You know it’s got loads of benefits for patients because you know you don’t get side effects from social prescribing.” Participant 6 (Employee, Independent)*

Pharmacistsreported the need to work with others who are already social prescribing to learn, share best practice and develop a common understanding.

“*Ultimately though this isn’t something pharmacies could just do on their own, we need to be linked up with other people doing this, like is there a national body of social prescribers or like standardised training about how to do it? If we knew more about social prescribers we would be linked in with that network more.” Participant 10 (Owner, Independent)*

Collectively this theme demonstrates complexity in pharmacists’ views of the outcomes of social prescribing, primarily being reliant on the social capital pharmacists have with patients and other staff in their premises but also on building social capital by engaging with other social prescribing networks and experts.

## Discussion and conclusion

### Summary of findings

The key finding of this study is participants appeared to recognise, understand and value social prescribing as a means of supporting patients’ health and well-being, but misunderstood social prescribing as a form of disease-focused, public health promotion. Limited training, experience and resources to facilitate social prescribing in practice were identified as learning needs in this study. Participants reported willing to be involved in social prescribing, reporting interests to better understand the process of social prescribing and expressing beliefs that this could expand the current role of community pharmacists and their team members. Many participants reported limited exposure to or involvement with social prescribing in current practice and education. This indicates a need for further collaboration and involvement in social prescribing networks. Professional bodies may also need to support education, learning and training of pharmacists and their teams to implement social prescribing services. The unique accessibility of community pharmacy teams and the rapport they have with their patients were seen as opportunities to contribute to social prescribing to improve patient outcomes.

A strength of the study is it provides a conceptualization of pharmacists’ understanding of social prescribing. The study met theoretical data sufficiency and used qualitative methods to identify insights. Additionally, the sample included pharmacists from a range of practice settings across North East England, which means the findings may be transferable to different contexts of practice. However, using convenience sampling meant these findings may not include the range of views across the pharmacy profession–particularly from those outside of North East England. Additionally, recruitment via social media introduces self-selection bias (whereby pharmacists with little interest in social prescribing would have been recruited) which may positively skew the findings in terms of participants’ reported willingness and enthusiasm for social prescribing rather than the reported limited exposure and understanding of it.

### Comparison to existing literature

The findings presented here add to the literature, demonstrating pharmacists are enthusiastic, but do not fully appreciate the scope and impact of social prescribing. The findings are congruent with a survey completed by 120 pharmacists, showing poor understanding of social prescribing, and the need for increased staff training and funding [29]. Existing literature has suggested pharmacists could adopt multiple roles to implement social prescribing–as screeners, identifiers, link workers or providers of social interventions [28, 39] to reduce the demand on existing health services [8]. However, with such a limited understanding shown in this research, the role pharmacists could adopt to implement social prescribing at present may be limited.

Some existing literature has stated that the impact of social prescribing may be overestimated [13]. A key reason for this, put forward by Gibson, Pollard (13), using a Bourdieuan lens, is focused on patients’ structural contexts; access to economic, social and cultural capital influences engagement with social prescribing interventions. Our study extends the argument from patients to pharmacists, highlighting that structural context also influences professional engagement with social prescribing interventions. Our study demonstrated that pharmacists have little cultural, economic and social capital to invest in social prescribing—their conceptualisation of it is limited (cultural capital), funding is poor and workload is high (economic capital) and their connections to professional social prescribing networks and bodies is poor (social capital) [40, 41]. This may hinder the capability, opportunity and motivation for pharmacists to engage in social prescribing. Further research such as feasibility and pilot studies, as well as trials, are needed to understand and consider the effectiveness of pharmacists and their teams bridging the gap between health and social care to help communities most in need.

### Implications for policy and practice

The NHS has made a commitment to increase social prescribing activity and expand the number of link workers [2, 42]. Pharmacists, with adequate economic, social and cultural capital, could support this—either by identifying patients for referral to link workers or providing link worker services ‘in house’ [28]. However, this study has shown that although pharmacists are interested in social prescribing, it appears to be positioned within current pharmacy practice as ‘healthy lifestyle changes’, ‘health promotion’ and ‘public health’ initiative, rather than supporting patients to deal with broader socioeconomic determinants of health, such as poor housing, economic hardship, and abusive relationships–which many link workers currently deal with through social prescribing [9, 10, 43]. If pharmacists are going to refer patients to social prescribing, then additional training, access and engagement with link workers will be needed to upskill the current workforce. Furthermore, establishing ways to build social connections of pharmacists with those involved in delivering social prescribing are required. If pharmacists themselves are going to act as link workers ‘in house’, then the findings suggest a much greater effort will be needed to enable them to have the skills, expertise, supervision and support structures to build their cultural capital to deal with non-clinical social issues to optimise health outcomes. Our findings show pharmacists believe they know what social prescribing is but their beliefs are not aligned to what social prescribing link workers actually do in reality. It shows there is a gap between pharmacists’ beliefs and social prescribing practice. This provides a very specific target for educators and policy makers to create an intervention to change pharmacists’ perceptions of social prescribing from a ‘healthy lifestyle intervention’ to a new praxis of ‘social pharmaceutical care’. This raises questions for policy makers and practitioners, and the profession as a whole–is social prescribing something community pharmacy teams want to do, given current high workloads in the sector?

## Conclusions

This study aimed to explore community pharmacists’ experiences of social prescribing. It has shown how they recognise and value social prescribing, but currently have limited understanding, training, experience and resources to incorporate it into their practice. These findings provide an insight into pharmacists understanding but may not be generalisable or transferable. Further work is therefore needed to explore if, when and how pharmacists and their teams could engage with social prescribing.
